# Redox-sensing regulator Rex regulates aerobic metabolism, morphological differentiation, and avermectin production in *Streptomyces avermitilis*

**DOI:** 10.1038/srep44567

**Published:** 2017-03-17

**Authors:** Xingchao Liu, Yaqing Cheng, Mengya Lyu, Ying Wen, Yuan Song, Zhi Chen, Jilun Li

**Affiliations:** 1 State Key Laboratory of Agro-biotechnology and MOA Key Laboratory of Soil Microbiology, College of Biological Sciences, China Agricultural University, Beijing 100193, China.

## Abstract

The regulatory role of redox-sensing regulator Rex was investigated in *Streptomyces avermitilis*. Eleven genes/operons were demonstrated to be directly regulated by Rex; these genes/operons are involved in aerobic metabolism, morphological differentiation, and secondary metabolism. Rex represses transcription of target genes/operons by binding to Rex operator (ROP) sequences in the promoter regions. NADH reduces DNA-binding activity of Rex to target promoters, while NAD^+^ competitively binds to Rex and modulates its DNA-binding activity. Rex plays an essential regulatory role in aerobic metabolism by controlling expression of the respiratory genes *atpIBEFHAGDC, cydA1B1CD, nuoA1-N1, rex*-*hemAC1DB, hppA*, and *ndh2*. Rex also regulates morphological differentiation by repressing expression of *wblE*, which encodes a putative WhiB-family transcriptional regulator. A *rex*-deletion mutant (Drex) showed higher avermectin production than the wild-type strain ATCC31267, and was more tolerant of oxygen limitation conditions in regard to avermectin production.

*Streptomyces*, a genus of filamentous Gram-positive soil-dwelling bacteria, are obligate aerobes^1^,^2^. During growth, they encounter a variety of environmental stresses resulting from the complex nature of soil. Growth in wet soil is particularly challenging because little or no oxygen is present. *Streptomyces* strains are widely used in industrial production of various antibiotics, and oxygen supply is a key parameter determining product yield during the antibiotic fermentation process^3^. It is important to understand how *Streptomyces* strains sense and respond to oxygen limitation.

Rex is a redox-sensing regulator widely distributed in Gram-positive bacteria^4^,^5^. NAD (H) plays a central role in redox metabolism. NAD^+^ is reduced to NADH by accepting electrons during substrate oxidation, and NADH is then reoxidized by the electron transport chain. Changes of oxygen status are reflected by the intracellular ratio of NADH to NAD^+^. When NADH/NAD^+^ ratio is low, Rex binds to target genes and represses transcription of genes involved in NAD^+^ regeneration. In contrast, high NADH/NAD^+^ ratio inhibits DNA-binding activity of Rex and derepresses transcription of its target genes^4^,^6^,^7^,^8^. Rex was first identified in *Streptomyces coelicolor* and shown to regulate expression of cytochrome *bd* terminal oxidase (*cydABCD* operon) and heme biosynthesis (*rex*-*hemACD* operon)^4^. In *Bacillus subtilis*, a facultative aerobe, Rex represses expression of cytochrome *bd* oxidase (*cydABCD*), NADH dehydrogenase (*yjlC-ndh*), NADH-linked fermentative lactate dehydrogenase (*lctP-ldh*), and a formate-nitrate transporter (*ywcJ*)^6^,^9^,^10^. In *Staphylococcus aureus*, Rex directly regulates at least 19 genes. It acts as a central regulator of anaerobic metabolism leading to anaerobic NAD^+^ regeneration, which includes lactate, formate, and ethanol fermentation (*adh1, adhE, lctP, ldh1, pflBA*) and nitrate respiration (*narG, nirC, nirR*)^7^. The binding sequence of Rex (Rex operator; ROP) is highly conserved in Gram-positive bacteria. Reported consensus sequence in *S. coelicolor* (5′-TGTGAACNNNTTCACA-3′)^4^, in *B. subtilis* (5′-WWTGTGAANTNNTNNNCAAW-3′; W represents either A or T)^10^, and in *S. aureus* (5′-TTGTGAAWWWWTTCACAA-3′)^7^ are very similar to the palindromic sequence in *S. coelicolor*.

Even though Rex was first characterized in *S. coelicolor* and its regulatory mechanism has been extensively studied, few target operons/genes of Rex in *Streptomyces* have been confirmed^4^, and the overall regulatory function of Rex in this genus remains to be elucidated. *S. avermitilis* is an important species used for industrial production of avermectins, a group of anthelmintic antibiotics widely used in the medical, veterinary, and agricultural fields^11^. We investigated the regulatory role of Rex in the expression of operons/genes involved in aerobic metabolism, morphology, and secondary metabolism of *S. avermitilis*. Our findings have potential application to novel genetic engineering strategies for high antibiotic-producing strains and hypoxia-tolerating strains of this genus.

## Results

### Expression of *atpIBEFHAGDC, cydA1B1CD, nuoA1-N1*, and *rex-hemAC1DB* is negatively regulated by Rex

**Figure 1:**
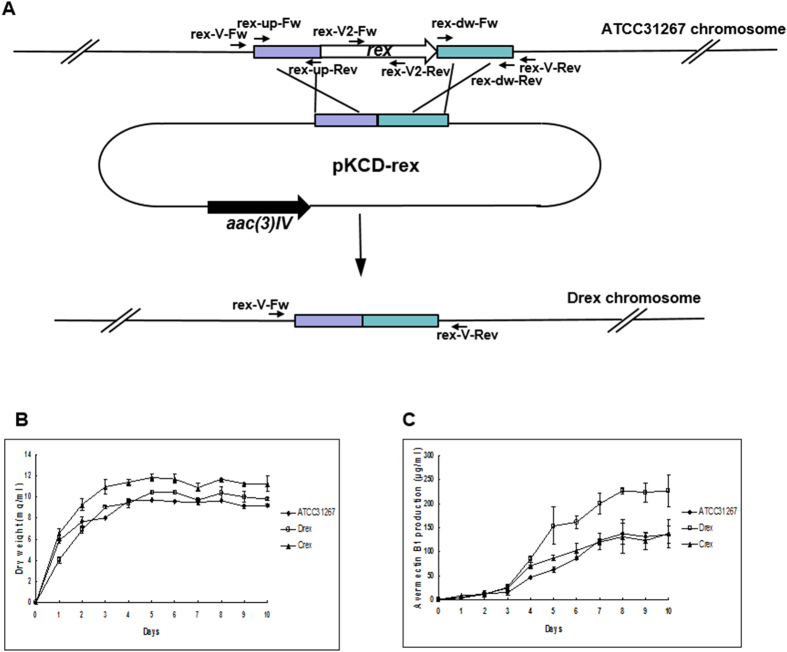
Construction and growth curve of *rex*-deletion mutant. (**A**) Method (schematic) used for *rex* deletion. Long arrows: genes and their directions. Short black arrows: positions of primers used for cloning of exchange regions and confirmation of gene deletion (see M&M). Rectangles: exchange regions used for *rex* deletion. (**B,C**) Growth (**B**) and avermectin production (**C**) of *rex-*related mutant strains in liquid fermentation medium II. Values shown are mean ± SD from three replicate flasks.

The *rex* gene is conserved within the genus *Streptomyces* and is cotranscribed with the heme synthesis genes *hemACD*^4^. To evaluate the regulatory role of Rex in *S. avermitilis*, we constructed a *rex*-deletion mutant (termed Drex) by homologous recombination in wild-type strain ATCC31267. *rex* deletion had no effect on growth in liquid fermentation medium (Fig. 1).

The promoter regions of the operons *atpIBEFHAGDC, cydA1B1CD, nuoA1-N1*, and *rex*-*hemAC1DB* in *S. avermitilis* all contain a putative Rex-binding motif, 5′-TTGTGAANNNNTTCACAA-3′ (Table 1). *nuoA1-N1* (SAV4837-4850) encodes putative NADH dehydrogenase I (complex I), *cydA1B1CD* (SAV4260-4258) encodes putative cytochrome *bd*-I oxidase (cytochrome *bd* complex), *atpIBEFHAGDC* (SAV2888-2880) encodes putative F-type proton-transporting ATPase, and *hemAC1DB* (SAV4739-4742) is cotranscribed with *rex* and encodes heme synthesis enzymes (Fig. S1). NADH dehydrogenase I, cytochrome *bd*-I oxidase, and F-type proton-transporting ATPase are essential components of the respiratory chain. Heme is most abundant in cytochromes, which are electron transfer proteins involved in the final reduction of oxygen during aerobic respiration. We performed qRT-PCR to determine whether expression of these genes involved in aerobic respiration is regulated by Rex.

**Figure 2:**
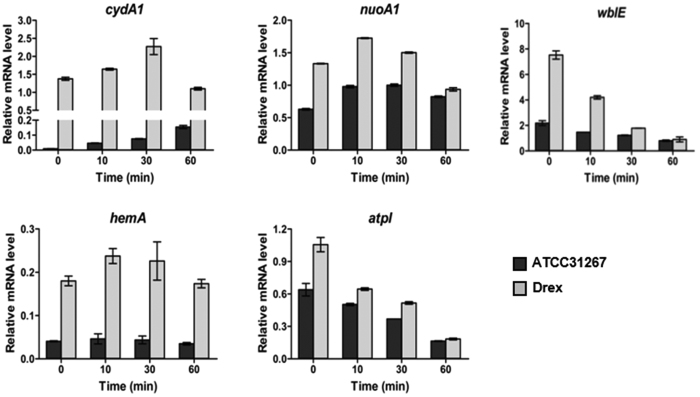
RT-qPCR analysis of transcription levels of *cydA1, nuoA1, hemA, atpI*, and *wblE* in ATCC31267 and Drex. RNA was prepared from cells grown in fermentation medium for 3 days on a rotary shaker (250 rpm) and then static-cultured for the indicated time. Quantitative data were normalized to *hrdB (SAV2444*) expression value. Values shown are mean ± SD from three replicates.

Expression of these genes differed greatly when cells were static-cultured following 3 days’ culture on a rotary shaker (250 rpm). Transcription level of *cydA1* under oxygen limitation condition in ATCC31267 increased steadily during 60 min, whereas the level in Drex increased to a maximal value during the first 30 min, then gradually declined during the subsequent 30 min (Fig. 2). These findings suggest that induction of *cydA1* under oxygen limitation condition is mediated by Rex. Expression of *nuoA1* and *hemA* under oxygen limitation increased slightly in the first 10 min, then declined during the subsequent 50 min, in both ATCC31267 and Drex. In contrast, expression of *atpI* under oxygen limitation declined steadily during 60 min in both ATCC31267 and Drex (Fig. 2). Transcription levels of *cydA1, nuoA1, hemA*, and *atpI* were consistently higher for Drex than for ATCC31267 under equivalent treatments, confirming that these genes are negatively regulated by Rex.

**Figure 3:**
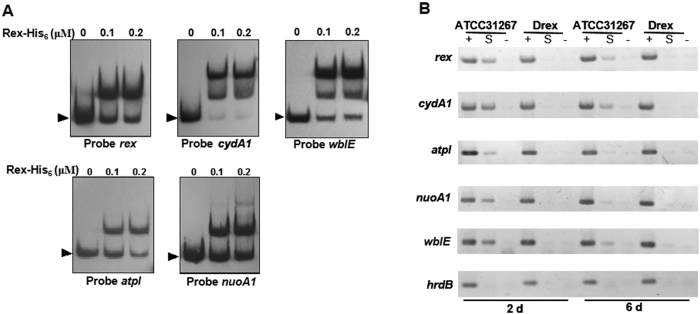
Binding of Rex-His_6_ to promoter regions of *rex, cydA1, atpI, nuoA1*, and *wblE.* (**A**) EMSAs using Rex-His_6_ protein at the indicated concentrations, and the probes indicated below the panels. Arrow: free probe. (**B**) ChIP assay analysis of Rex binding to promoter regions of *rex, cydA1, atpI, nuoA1*, and *wblE in vivo*. Rex-DNA complexes were immunoprecipitated by anti-Rex antibodies from formaldehyde-treated ATCC31267 and Drex cells. DNAs used for PCR were: total DNA prior to immunoprecipitation (positive control: lanes “+”), immunoprecipitated DNA (experimental sample: lanes “S”), and DNA without antibody (negative control: lanes “−”). *hrdB* promoter region was used as control.

C-terminal His_6_-tagged Rex fusion protein was overexpressed in *E. coli* and purified for DNA binding analysis. EMSAs were performed to evaluate interactions between Rex and the promoters *in vitro*. Rex-His_6_ bound to the promoter regions of *atpIBEFHAGDC, cydA1B1CD, nuoA1-N1*, and *rex-hemAC1DB* operons (Fig. 3A). ChIP assays were performed to assess interactions *in vivo*. ATCC31267 and Drex cells were treated with formaldehyde at days 2 and 6 to cross-link Rex to its DNA targets. Cross-linked DNA was extracted, fragmented by sonication, and immunoprecipitated by anti-Rex antibodies for screening of Rex-bound DNA fragments. In comparison to control *hrdB* promoter, PCR products of *rex, cydA1, atpI*, and *nuoA1* promoter regions were selectively enriched from immunoprecipitated DNA of ATCC31267, whereas no such PCR bands were amplified from immunoprecipitated DNA of Drex (Fig. 3B). Results of EMSAs and ChIP assays revealed that Rex binds specifically to the promoter regions of *atpIBEFHAGDC, cydA1B1CD, nuoA1-N1*, and *rex-hemAC1DB* operons.

### Determination of Rex operator (ROP) sequences on promoter regions of *atpIBEFHAGDC, cydA1B1CD, nuoA1-N1*, and *rex-hemAC1DB*

Rex binding sequences in 5′-end fluorescein-labeled promoter regions of the above operons were determined by DNase I footprinting analysis. One protected region was detected in the *rex* promoter region in the presence of 1.2 or 2.4 μΜ Rex-His_6_. The region extends for 23 nucleotides from positions −39 to −17 relative to the transcriptional start site (TSS) of *rex*. A consecutive ROP site (5′-**TTGTGCA**CGCG**TTCACAA**-3′) was found in the protected region; the site is located between −35 region and −10 region and encompasses −35 region (Fig. S2A). A 28-nt protected region (positions −3 to + 25 relative to TSS) was detected in the *cydA1* promoter region. One ROP site (5′-**ATGTGAA**CGCG**TTCACAA**-3′) was found in the protected region downstream from TSS. A half-site ROP (5′-**TTGTGAA**-3′) was also found in the protected region; it is located upstream from the ROP site and encompasses TSS (Fig. S2B). EMSA revealed two retarded bands between Rex and the *cydA1* promoter region (Fig. 3A), suggesting that Rex can interact with the half-site ROP. A 29-nt protected region (positions −50 to −22 relative to TSS) containing a ROP site (5′-**TTGTGAT**ACGG**TTCACGA**-3′) was detected in the *atpI* promoter region (Fig. S2C). Rex-His_6_ protected a 27-nt region extending from positions −42 to −16 relative to TSS of *nuoA1*, which contains a ROP site (5′-**TTGTGAC**CTGC**TTCACAT**-3′) (Fig. S2D). ROP in the *nuoA1* and *atpI* promoter regions is located between −35 region and −10 region, and encompasses −35 region. These findings suggest that Rex blocks attachment of RNA polymerase to the promoters or inhibits the progress of RNA polymerase by binding to ROP in or downstream of the promoters of *atpIBEFHAGDC, cydA1B1CD, nuoA1-N1*, and *rex-hemAC1DB*, and blocks transcription of these operons.

### DNA-binding activity of Rex is modulated by NADH/NAD^+^ ratio

**Figure 4:**
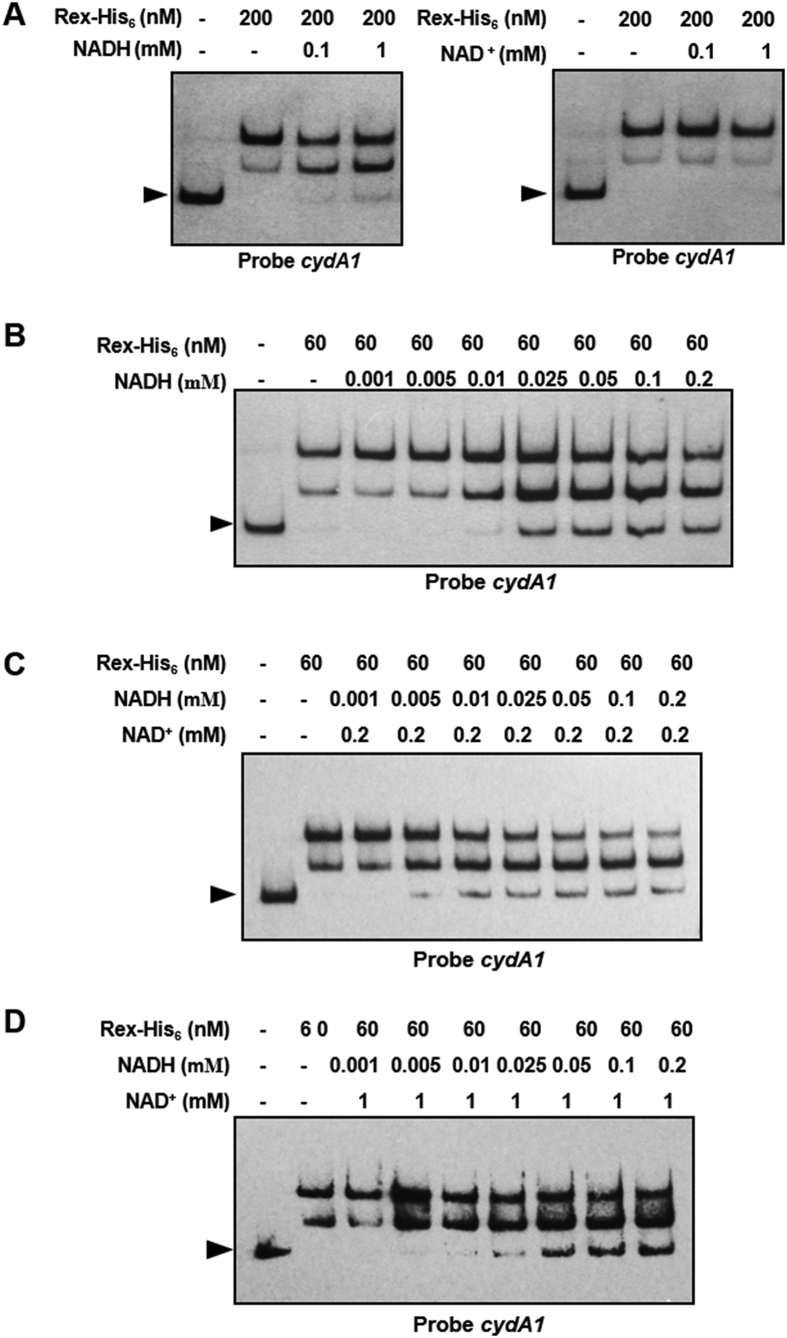
DNA-binding activity of Rex is modulated by NADH/NAD^+^ ratio. (**A**) EMSAs of *cydA1* promoter region using Rex-His_6_ and 0.1 or 1 mM pyridine nucleotides. (**B**) EMSAs of *cydA1* promoter region using Rex-His_6_ with various NADH concentrations. (**C**,**D**) Assay mixtures contained NADH at indicated concentration and 0.2 mM (**C**) or 1 mM (**D**) NAD^+^. Arrow: free probe.

In *S. coelicolor*, NADH at concentrations <5 μM inhibits DNA-binding activity of Rex, whereas 1 mM NAD^+^ has no inhibitory effect. NAD^+^ competes with NADH for Rex binding^4^. In *B. subtilis* and *S. aureus*, NAD^+^ enhances binding of Rex to putative Rex-binding sites, while NADH competes with NAD^+^ for Rex binding and reduces Rex activity^6^,^7^. We examined the effects of NAD^+^ and NADH on DNA-binding activity of Rex to upstream regions of *cydA1* in *S. avermitilis*. DNA-binding activity of Rex was reduced by addition of NADH, but not by NAD^+^ concentrations up to 1 mM (Fig. 4A,B; Fig. S3). NADH and NAD^+^ were added to EMSA binding buffer to assess the effect of NAD^+^/NADH ratio on DNA-binding activity of Rex *in vitro*. At NAD^+^ concentration 0.2 mM, 5 μM NADH was sufficient to dissociate the Rex-DNA complex (Fig. 4C). At NAD^+^ concentration 1 mM, dissociation of DNA-Rex complex required 25 μM NADH, suggesting that Rex-binding activity was recovered by addition of increasing amounts of NAD^+^ (Fig. 4D). These findings indicate that NAD^+^ and NADH bind competitively to Rex and modulate its DNA-binding activity. These findings also imply that Rex exploits the similar regulatory mechanism in *Streptomyces*.

### Rex regulates morphological differentiation

**Figure 5:**
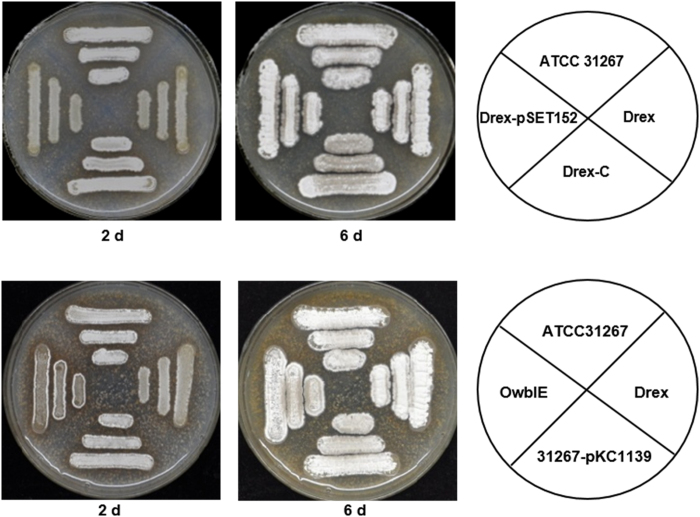
Phenotypes of *rex*- and *wblE*-related mutant strains. Growth of indicated strains on SFM agar for 2 and 6 days. WT, wild-type ATCC31267; Drex, *rex-*deletion mutant; Drex-C, complementation strain of Drex; Drex-pSET152 and 31267-pKC1139, empty plasmid-containing controls; OwblE, *wblE* overexpressing strain.

In comparison to ATCC31267, Drex showed delayed morphogenesis on SFM agar at day 2, when aerial mycelium was initiated. Spore formation at day 6 did not differ notably between the two strains. Morphogenesis of the Drex complementation strain was similar to that of ATCC31267 (Fig. 5), indicating that the delayed morphogenesis was due solely to *rex* deletion.

The promoter region of *wblE* in *S. avermitilis* contains a putative Rex-binding motif (Table 1). *wblE* encodes a putative WhiB-family transcriptional regulator, which may be involved in morphological differentiation^12^,^13^. qRT-PCR analysis revealed notable increases of *wblE* transcription level in Drex. Levels under oxygen limitation condition declined gradually during 60 min for both ATCC31267 and Drex, and were consistently higher for Drex than for ATCC31267 (Fig. 2). EMSAs showed that Rex-His_6_ bound to the *wblE* promoter region *in vitro* (Fig. 3A). In *in vivo* ChIP assays, PCR product of the *wblE* promoter region was selectively enriched from immunoprecipitated DNA of ATCC31267, whereas no such PCR band was amplified from immunoprecipitated DNA of Drex (Fig. 3B). These findings indicate that *wblE* is negatively regulated by Rex. Rex binding sequence in the *wblE* promoter region was determined by DNase I footprinting analysis. A 28-nt region protected by Rex-His_6_ was detected, extending from positions + 108 to + 135 relative to TSS of *wblE* (Fig. S4). The protected region contains a consecutive ROP site (5′-**TCGTGAA**AGCG**TTCACAT**-3′) and a half-site ROP (5′-**TTCACAA**-3′) located downstream of TSS. Rex may inhibit the progress of RNA polymerase by binding to ROP downstream of the *wblE* promoter, and thereby repress transcription.

To test the possibility that overexpression of *wblE* in Drex results in delayed morphogenesis, we attempted to delete *wblE* in *S. avermitilis*. However, this attempt was unsuccessful. *wblE* is evidently an essential gene in *Streptomyces*; an attempt to delete it in *S. coelicolor* was also unsuccessful^13^. When *wblE* was overexpressed in ATCC31267, the resulting strain had a phenotype similar to that of Drex (Fig. 5), suggesting that Rex regulates morphological differentiation through its effect on *wblE* expression.

### Rex negatively regulates avermectin production

**Figure 6:**
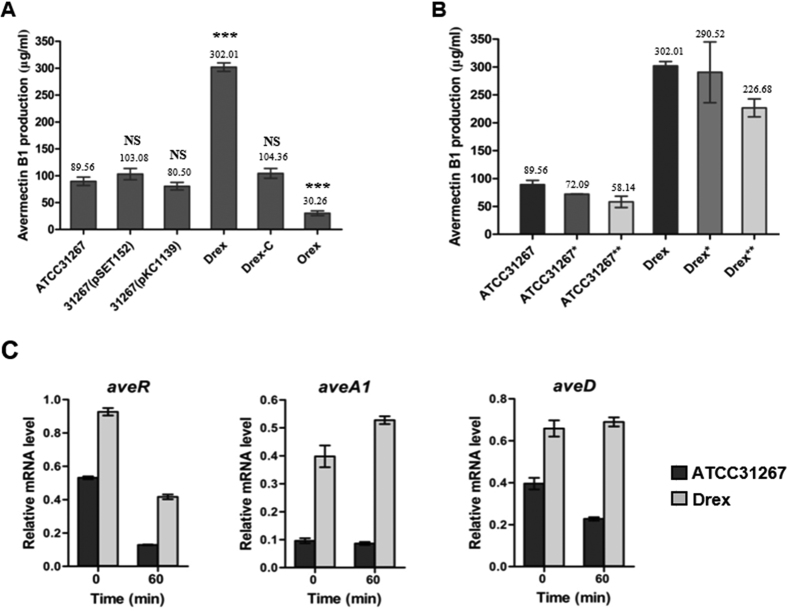
Effect of *rex* deletion on avermectin production in *S. avermitilis.* (**A**) Avermectin production in *rex*-related mutant strains. ATCC31267, wild-type; Drex, *rex*-deletion mutant; 31267 (pSET152) and 31267 (pKC1139), empty plasmid-containing controls; Drex-C, *rex*-deletion complementation strain 31267 (pSET-rex); Orex, *rex* overexpressing strain 31267 (pKC-rex). (**B**) Avermectin production in ATCC31267 and Drex with various oxygen limitation conditions. *Agitation speed 230 rpm (control, 250 rpm); **Static culture for 2 h at day 5 during fermentation (250 rpm). (**C**) RT-qPCR analysis of *aveR, aveA1,* and *aveD* transcription levels in ATCC31267 and Drex. RNA samples were the same ones used for experiments shown in Fig. 1. Quantitative data were normalized to *hrdB* expression value. Values shown are mean ± SD from three replicates. Statistical significance of differences was determined using Student’s *t*-test. ***P < 0.001; NS, not significant.

The overexpression of *rex* caused a decrease in avermectin production to 33% of ATCC31267 level. Drex had avermectin production ~3-fold higher than that of ATCC31267. The mycelial dry weight of Drex was similar to that of ATCC31267, indicating that the improved avermectin yield was not achieved by improved growth. In the Drex complementation strain, avermectin production was similar to that of ATCC31267 (Fig. 1; Fig. 6A). These findings indicate that *rex* negatively regulates avermectin production in *S. avermitilis*. We also measured avermectin production under oxygen limitation condition. In ATCC31267, lower agitation speed (230 rpm; control speed was 250 rpm) resulted in a 20% reduction of avermectin production, and static culture for 2 h on day 5 resulted in a 35% reduction. In Drex, avermectin production was reduced by 3.8% and 25%, respectively, under the above two conditions (Fig. 6B). Thus, *rex* deletion resulted in increased tolerance of *S. avermitilis* to oxygen limitation in regard to avermectin production.

**Figure 7:**
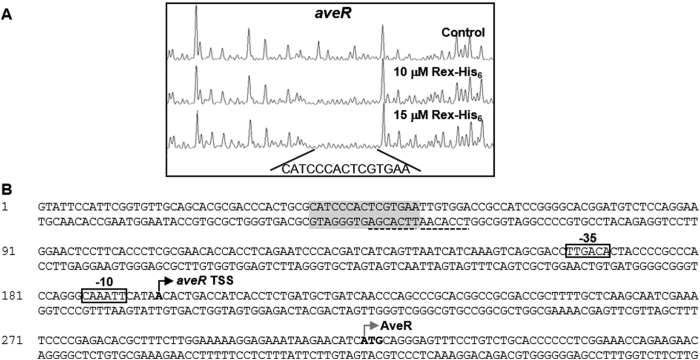
Determination of Rex binding site on *aveR* promoter region by DNase I footprinting assay. (**A**) Fluorograms correspond to control DNA fragment and to protected reactions (with 10 and 15 µM Rex-His_6_). (**B**) Nucleotide sequences of *aveR* promoter region. Shaded boxes: sequences protected from DNase I digestion. Dotted boxes: ROP. Dotted line: half-site ROP. Black bent arrows with boldface letters: TSSs. Boxes: presumed −35 and −10 elements of promoters. Gray bent arrows with boldface letters: translational start codons.

qRT-PCR analysis was performed to determine whether Rex regulates avermectin production at the transcriptional level. Drex showed significantly increased transcription levels of pathway-specific regulatory gene *aveR* and biosynthetic genes *aveA1* and *aveD*, relative to ATCC31267. Oxygen limitation for 60 min reduced expression of these genes in ATCC31267; however, Drex showed lower fold repression, and a slight induction of *aveA1* and *aveD* (Fig. 6C). EMSAs revealed that Rex-His_6_ did not bind to the *aveR* promoter region or the *aveD-A1* intergenic region (Fig. S5). Although no retarded band was observed when *aveR* promoter region was probed with Rex-His_6_ protein, DNase I footprinting analysis showed one protected region extending for 15 nucleotides on the *aveR* coding strand in the presence of 10 or 15 μΜ Rex-His_6_ (Fig. 7). No consecutive ROP site was observed in the protected region; however, two adjacent half-site ROP (5′-**TCGTGAA**-3′ and 5′- **TTGTGGA**-3′) were found in the protected region and downstream region. Rex can evidently interact with the half-site ROP; however, because the interaction is weak and easily dissociated *in vitro*, EMSA did not reveal a clear shifted band.

### Confirmation of putative Rex target genes

**Figure 8:**
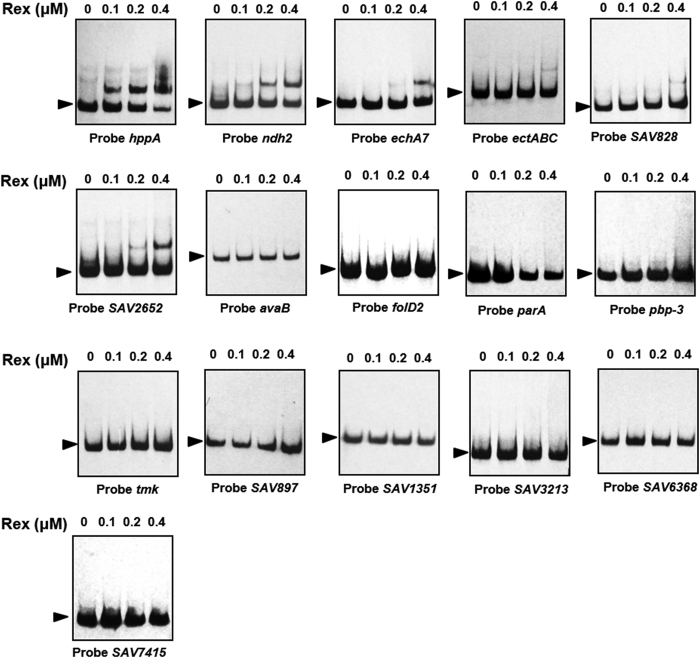
Binding of Rex-His_6_ to promoter regions of putative Rex targets. Rex (μM): Rex-His_6_ concentrations. Arrow: free probe.

A genome-wide search of consensus motif 5′-TTGTGAANNNNTTCACAA-3′ using the genome sequence of ATCC31267 revealed the presence of 36 motifs up to 350 bp upstream of predicted genes: 2 motifs with one mismatch, 10 motifs with two mismatches, and 24 motifs with three mismatches. Our previous experiments showed that *wblE, cydA1B1CD, rex-hemAC1DB, atpIBEFHAGDC*, and *nuoA1-N1* are directly controlled by Rex. To investigate whether Rex binds to promoter regions of other putative target genes, we selected 16 genes with predicted gene function for EMSAs (Table 1). Of these, Rex bound to the probes of *hppA* (encodes an inorganic H^+^ pyrophosphatase), *ndh2* (encodes a NADH dehydrogenase [complex I]), *echA7* (encodes an enoyl-CoA hydratase), *ectABC* (encodes ectoine biosynthesis enzymes), *SAV828* (encodes a rhamnosidase), and *SAV2652* (encodes a regulatory protein). Probes whose binding motif had one or two mismatches showed higher affinity than probes whose binding motif had three mismatches (Fig. 3A, Fig. 8). These findings demonstrated that 5′-TTGTGAANNNNTTCACAA-3′ is the consensus motif of Rex in *S. avermitilis*.

## Discussion

**Figure 9:**
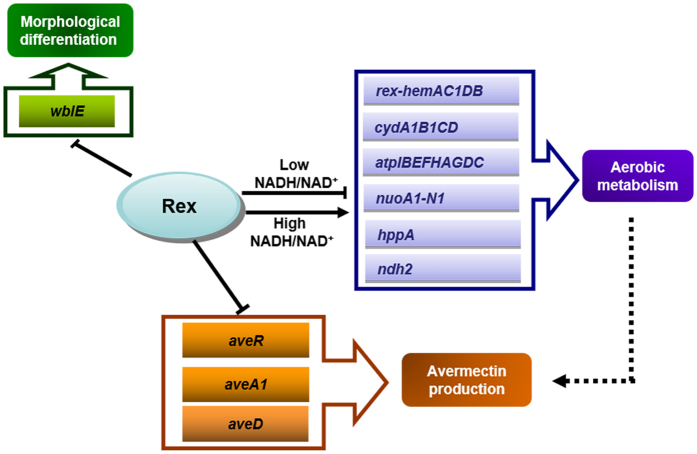
Regulatory network (schematic) whereby Rex controls major genes involved in aerobic metabolism, morphological differentiation, and secondary metabolism. Solid arrows: direct induction. Solid lines with blunt end: repression. Dashed arrows: indirect induction.

Results of this study show that Rex in *S. avermitilis* acts as a repressor of aerobic metabolism, morphological differentiation, and secondary metabolism (summarized schematically in Fig. 9). Results of EMSAs demonstrated that at least 11 genes/operons are directly regulated by Rex. Among these, *atpIBEFHAGDC, cydA1B1CD, nuoA1-N1*, and *rex*-*hemAC1DB* operons encode key components of the electron transfer chain and play crucial roles in aerobic metabolism^14^,^15^,^16^,^17^. *hppA* encodes a putative pyrophosphate-energized proton pump that converts energy from pyrophosphate hydrolysis into active H^+^ transport across the plasma membrane^18^. *ndh2* encodes a NADH dehydrogenase involved in NAD^+^ regeneration^19^,^20^. *echA7* encodes an enoyl-CoA hydratase that catalyzes the second step of the β-oxidation pathway of fatty acid metabolism^21^. *SAV828* encodes a rhamnosidase that hydrolyzes L-rhamnose from L-rhamnoside^22^. Under oxygen limitation condition, the increase of intracellular NADH/NAD^+^ ratio in *S. avermitilis* dissociates binding of Rex from its target binding sites and derepresses its target genes/operons, and upregulation of *cydA1B1CD, nuoA1-N1, rex*-*hemAC1DB, ndh2,* and *hppA* increases oxygen utilization, NAD^+^ regeneration, and ATP synthesis (Fig. 9). On the other hand, expression of *atpIBEFHAGDC* in ATCC31267 and Drex is downregulated by oxygen limitation, suggesting that this operon is also directly controlled by regulators other than Rex. The F_0_F_1_-ATPase operon in *Corynebacterium glutamicum* is regulated by ECF σ^H ^23. A *sigH* homolog is present in *Streptomyces*; whether it regulates *atpIBEFHAGDC* expression remains to be tested.

WhiB-like family transcription factors are widely present in actinomycetes, but not found in other bacterial orders. WhiB was first identified as a small transcription factor-like protein essential for sporulation in *S. coelicolor*^24^. Genome sequencing revealed that *Streptomyces* species have multiple *whiB*-like genes (designated “*wbl*”). Eleven *wbl* genes (including *whiB* and *whiD*) have been identified in *S. coelicolor*^13^. Among these, *wblA, whiB*, and *whiD* are essential for sporulation, and WblA also negatively regulates antibiotic biosynthesis in *Streptomyces*^13^,^25^,^26^,^27^. Other *wbl* genes are not involved in morphological development, with the exception of *wblE*. Fowler-Goldsworthy *et al*.^13^ reported that *wblE* could not be deleted in various strains of *S. coelicolor*, and we made a similar observation in *S. avermitilis*. Thus, *wblE* appears to be essential in this genus. The homolog of *wblE* in *Mycobacterium tuberculosis* is *whiB1*, which encodes an essential transcription factor in response to nitric oxide exposure^28^. We demonstrated that *wblE* is directly negatively regulated by Rex, and that *wblE* overexpression results in delayed morphogenesis similar to that of Drex. Expression of *wblE*, like that of *atpIBEFHAGDC*, is downregulated by oxygen limitation in both ATCC31267 and Drex, suggesting that (i) *wblE* is jointly regulated by Rex and some other regulator, or (ii) *wblE* itself responds to low oxygen concentration via its own redox-sensitive [4Fe-4S] cluster. Under oxygen limitation condition, *wblE* expression in *Streptomyces* is downregulated, with consequent stimulation of sporulation and production of a large number of spores to maintain viability under conditions of little or no oxygen. Another Rex target, *ectABC*, encodes enzymes for biosynthesis of ectoine (a compatible solute) that serves as an osmolyte and promotes survival under osmotic or temperature stress^29^. By regulating *ectABC* transcription, Rex facilitates ectoine biosynthesis to enhance viability under these types of stress.

In Drex, expression of regulatory gene *aveR* and biosynthetic genes, and avermectin production, were notably increased. Although EMSA showed no clearly retarded band between *aveR* promoter region probe and Rex-His_6_, DNase I footprinting analysis revealed one 15-nt protected region consisting of two adjacent half-site ROP on the coding strand of *aveR* by Rex-His_6_. Thus, Rex may directly regulate *aveR* expression by interacting with the half-site ROP in the *aveR* promoter region. Expression of electron transfer chain components was enhanced in Drex, thus promoting aerobic respiration rate, ATP production, and secondary metabolism. The notable increase of *atpIBEFHAGDC, cydA1B1CD, nuoA1-N1*, and *rex*-*hemAC1DB* expression in Drex enhanced the tolerance of cells to oxygen limitation. The findings described here provide a basis for construction of new *Streptomyces* strains with high antibiotic production and hypoxia tolerance.

## Materials and Methods

### Bacterial strains and growth conditions

The *S. avermitilis* strains used were ATCC31267 (wild-type), Drex (*rex*-deletion strain), Drex-C (*rex*-deletion complementary strain harboring plasmid pSET-rex), and Orex (ATCC31267 harboring *rex* overexpressing plasmid pKC-rex). *E. coli* strains JM109 and BL21 (DE3) were used for routine cloning and protein expression, respectively. YMS medium and SFM medium were used for sporulation and phenotype studies^30^,^31^. Culture conditions for mycelial growth, protoplast preparation, and regeneration of *S. avermitilis* were as described previously^30^. Seed medium and fermentation medium FM-I were used for avermectin production and for RNA isolation, and soluble fermentation medium FM-II was used for ChIP analysis^32^.

### Gene deletion, complementation, and overexpression

A *rex* (SAV4738) gene deletion mutant was generated through targeted gene deletion mediated by homologous recombination. A 566-bp fragment upstream of *rex* (position −460 to + 87 from start codon) was amplified by primers rex-up-Fw and rex-up-Rev, and a 579-bp fragment downstream of *rex* (position +539 to +1098) was amplified by primers rex-dw-Fw and rex-dw-Rev, using ATCC31267 genomic DNA as template (Table S1; Fig. 1). The two fragments, after recovery, were digested respectively by *Bam*HI/*Hin*dIII and *Bam*HI/*Eco*RI, and ligated together into *Eco*RI/*Hin*dIII-digested pKC1139^33^ to produce *rex*-deletion vector pKCD-rex. pKCD-rex was introduced into ATCC31267 protoplasts. Double-crossover recombinant strains were selected as described previously^34^,^35^. The *rex*-deletion mutant (termed Drex) was confirmed by PCR using one pair of external primers (rex-V-Fw/rex-V-Rev) and one pair of internal primers (rex-V2-Fw/rex-V2-Rev) (Table S1; Fig. 1). Use of the external primers yielded a 1.3-kb band from Drex and a 1.8-kb band from ATCC31267. Use of the internal primers yielded a 225-bp band from ATCC31267 and no band from Drex (data not shown).

A 1038-bp DNA fragment carrying the *rex* ORF and its putative promoter was amplified by PCR using primers rex-E-Fw and rex-E-Rev (Suppl. Table 1), and then ligated into *Eco*RI/*Xba*I-digested pSET152 or pKC1139 to produce vector pSET-rex or pKC-rex. For complementation analysis of Drex, pSET-rex was transformed into Drex protoplasts. For overexpression of Rex, pKC-rex was introduced into ATCC31267 protoplasts.

### RNA extraction and qRT-PCR analysis

RNA was isolated using Trizol reagent (Tiangen; China) from *S. avermitilis* mycelia grown in FM-I as described previously^32^. Transcription levels of various genes were determined by qRT-PCR using the primer pairs listed in Table S1. An RNA sample without prior reverse transcription was used as negative control to rule out chromosomal DNA contamination. *hrdB* gene (*SAV2444*) was used as internal control.

### Chromatin Immunoprecipitation (ChIP) assay

ChIP assay was performed as described previously^36^. In brief, *S. avermitilis* cultures grown in FM-II for 2 or 6 days were fixed in cross-linking buffer (0.4 M sucrose, 10 mM Tris·Cl [pH 8.0], 1 mM EDTA) containing 1% formaldehyde for 20 min at 28 °C. ChIP was performed using anti-Rex antibody. After DNA extraction, pellets were washed with 70% ethanol and resuspended in 50 μl Tris·EDTA buffer. 1 μl DNA solution was subjected to PCR using the primer sets listed in Table S1.

### Overexpression and purification of Rex-His_6_

The *rex* coding region was amplified by PCR using primers His-rex-Fw and His-rex-Rev. The purified fragment was cut with *Nco*I/*Hin*dIII and cloned into *Nco*I/*Hin*dIII-digested pET28a (+) to generate expression plasmid pET-rex. pET-rex was introduced into *E. coli* BL21 (DE3) for overexpression of C-terminal His_6_-tagged Rex. Rex-His_6_ was induced by 0.2 mM IPTG at 37 °C and purified from whole-cell lysate by Ni-NTA agarose chromatography (Bio-works; Sweden) according to the manufacturer’s instructions.

### Electrophoretic mobility gel shift assays (EMSAs)

EMSAs were performed according to the manufacturer’s instructions (DIG Gel Shift Kit, 2^nd^ Generation, Roche) as described previously^35^. DNA probes were obtained by PCR using the primers listed in Table S1, and labeled with Digoxigenin-11-ddUTP at the 3′ end using recombinant terminal transferase. DIG-labeled DNA probe was incubated with various quantities of Rex-His_6_ for 30 min at 25 °C in a total volume of 20 μl containing 1 μg poly[d(I-C)]. Electrophoresis (5.0% native polyacrylamide gel; 0.5 × TBE as running buffer) was performed to separate protein-bound probes from free probes. DNA was electroblotted onto a positively charged nylon membrane, and retarded and unbound bands were detected by chemiluminescence and recorded on X-ray film.

### DNase I footprinting assays

A fluorescent labeling procedure was used for DNase I footprinting assays^37^. DNA fragments were obtained by PCR using FAM-labeled primers (Table S1), and purified from agarose gel. Labeled DNA fragments (400 ng) and various quantities of Rex-His_6_ were incubated in a 25-μl volume for 30 min at 25 °C. DNase I digestion was performed for 40 sec at 37 °C, and terminated by addition of 10 μl 0.2 M EDTA (pH 8.0). Samples were subjected to phenol/chloroform extraction, ethanol precipitation, and capillary electrophoresis. Electrophoregrams were analyzed using GeneMarker software v2.2.0.

### Fermentation and HPLC analysis of avermectin production

Fermentation of *S. avermitilis* strains and estimation of avermectins yields by HPLC analysis were performed as described previously^32^.

### Determination of transcriptional start sites

Transcriptional start sites (TSS) of *rex* and *wblE* were mapped by 5′-RACE using a 5′/3′ RACE Kit (2nd Generation, Roche). Total RNA was extracted from ATCC31267 grown in FM-I for 2 days. A gene-specific primer (sp1) was used to synthesize cDNA, and template RNA was degraded with RNase H. A homopolymeric A-tail was purified and added to the 3′-end of cDNA using terminal transferase. Tailed cDNA was PCR amplified through 35 cycles with a specific nested primer (sp2) and an oligo (dT)-anchor primer (Table S1). PCR products were electrophoresed, purified using a DNA agarose gel recovery kit (BioTek; China), and sequenced.

### Prediction of Rex putative targets

To search for putative Rex target genes, Rex consensus motif 5′-TTGTGAANNNNTTCACAA-3′ was used to scan the intergenic regions of the *S. avermitilis* genome using Virtual Footprint software^38^.

## Additional Information

**How to cite this article:** Liu, X. *et al*. Redox-sensing regulator Rex regulates aerobic metabolism, morphological differentiation, and avermectin production in *Streptomyces avermitilis. Sci. Rep.* **7**, 44567; doi: 10.1038/srep44567 (2017).

**Publisher's note:** Springer Nature remains neutral with regard to jurisdictional claims in published maps and institutional affiliations.

## Supplementary Material

Supplementary Information

## Figures and Tables

**Table 1:** Putative Rex target genes.

Gene	Function	Nucleotide position^a^		PMW Score^b^	Sequence	ATG Distance^c^
		Start	End			
*cydA1B1CD (SAV4260-4258*)	putative cytochrome *bd*-I oxidase (cytochrome *bd* complex)	5226395	5226412	1.00	ATGTGAACGCGTTCACAA	81
*rex-hemAC1DB (SAV4738-4742*)	redox-sensing transcriptional repressor; heme biosynthetic enzymes	5776959	5776976	1.00	TTGTGCACGCGTTCACAA	77
*atpIBEFHAGDC (SAV2888-2880*)	putative F-type proton-transporting ATPase	3533332	3533349	2.00	TTGTGATACGGTTCACGA	137
*wblE (SAV3016*)	putative WhiB-family transcriptional regulator	3771378	3771395	2.00	ATGTGAACGCTTTCACGA	43
*hppA (SAV4616*)	putative inorganic H^+^ pyrophosphatase	5632612	5632629	2.00	TCGTGAATCAATTCACGA	195
*nuoA1-N1 (SAV4837–4850*)	putative NADH dehydrogenase I (complex I)	5880239	5880256	2.00	ATGTGAAGCAGGTCACAA	147
*pbp3-4 (SAV3603-SAV3604*)	putative penicillin-binding protein	4460250	4460267	2.00	TTCTGAACGTGTTCAGAA	37
*SAV828*	putative rhamnosidase	982811	982828	3.00	CTGTGAATCGATTCACCT	137
*echA7 (SAV2316*)	putative enoyl-CoA hydratase	2820643	2820660	3.00	TCGTGACGACAGTCACAA	66
*SAV2652*	putative regulatory protein	3252402	3252419	3.00	TTGTGCACCGCTTCACCC	288
*ndh2 (SAV3529*)	putative NADH dehydrogenase (complex I)	4369478	4369495	3.00	TTGTGAAGGGGCGCACGA	119
*ectABCD (SAV6398-6395*)	putative L-2,4-diaminobutyrate acetyltransferase, ectoine biosynthesis	7673586	7673603	3.00	TTGTGATCGACTCCACAT	155
*SAV6368*	putative multiple sugar ABC transporter permease protein	7638483	7638500	3.00	TGGTGAAGCGCTTCGCGT	81
*avaB- avaL2 (SAV2267-2268*)	putative gamma-butyrolactone- dependent transcriptional regulator	2766273	2766290	3.00	TCGTGAACGAATTCTAAT	29
*SAV3213*	putative nitroreductase family protein, NADH dehydrogenase/NAD(P)H nitroreductase	4004553	4004570	3.00	TGGTGATCGGCTTCACAG	96
*SAV1351*	putative fatty acid-CoA racemase	2820643	2820660	3.00	TCGTGACGACAGTCACAA	66
*tmk (SAV4622*)	putative thymidylate kinase	5643744	5643761	3.00	GTGTGGAGGCGTCCACAA	73
*folD2 (SAV543*)	putative methylenetetrahydrofolate	688370	688387	3.00	TTGTGTGTGAGTTCAGAA	231
*parA (SAV6508*)	putative partitioning or sporulation protein	7797460	7797477	3.00	ATGTCGACTCATTCACAA	114
*SAV7415-7416*	putative sugar isomerase putative simple sugar ABC transporter	8846427	8846444	3.00	TCGTGAAAGGTTTCAACT	203
*SAV897*	putative alpha-amylase inhibitor	1075479	1075496	3.00	TTGCGAAAGTTGTCGCAA	73

^a^Genomic position.

^b^Number of mismatches with respect to the consensus. PWM, positive weight matrix.

^c^Values are distances (in nucleotides) to the predicted start codon of the downstream gene.
