# Supplementary material for: Redox-sensing regulator Rex regulates aerobic metabolism, morphological differentiation, and avermectin production in Streptomyces avermitilis
Source: Sci Rep. 2017 Mar 17;7:44567. doi: 10.1038/srep44567 (PMC5355995; doi:10.1038/srep44567)
Supplement: Supplementary Information [file srep44567-s1.pdf]

# Redox-sensing regulator Rex regulates aerobic metabolism, morphological differentiation, and avermectin production in *Streptomyces avermitilis*

Xingchao Liu, Yaqing Cheng, Mengya Lyu, Ying Wen, Yuan Song, Zhi Chen, & Jilun Li

State Key Laboratory of Agro-biotechnology and MOA Key Laboratory of Soil Microbiology, College of Biological Sciences, China Agricultural University, Beijing 100193, China. Correspondence and requests for materials should be addressed to Z.C. (email: chenzhi@cau.edu.cn)

## Supplementary Table 1. Primers used in this study

| Purpose | Primer | Sequence (5'--3')[a] |
|---|---|---|
| For construction of Drex mutant | | |
| | rex-up-Fw | CCCAAGCTTCATCACCCAGGACGCGGAG (*Hin*dIII) |
| | rex-up-Rev | CGCGGATCCCTCGGAGGTACAGCGGAAGC (*Bam*HI) |
| | rex-dw-Fw | CGCGGATCCTCACCTCCATCCTGAACTTCG (*Bam*HI) |
| | rex-dw-Rev | CCGGAATTCCCCTCGCCCACGACCATC (*Eco*RI) |
| | rex-V-Fw | GGTGAGAATGGCCGACATGAGT |
| | rex-V-Rev | AACAGGTCGTTGAGGAGCCGT |
| | rex-V2-Fw | TGTGGGCTACGACGTCGAG |
| | rex-V2-Rev | AGCTCGTCCGTGTGCTGG |
| For complementation of Drex mutant | | |
| | rex-E-Fw | CGGAATTCGGAACGAAGGCGCCTGTGT (*Eco*RI) |
| | rex-E-Rev | GCTCTAGACTGGAGCAGCTTGACCTGG (*Xba*I) |
| For overexpression of *wblE* | | |
| | wblE-E-Fw | CGGAATTCGAGTCTCTACGTGGCGATCG (*Eco*RI) |
| | wblE-E-Rev | GCTCTAGACCAAGCTCAGGCTCGTTGC (*Xba*I) |
| For construction of His-tagged Rex | | |
| | His-rex-Fw | CATGCCATGGCAACTGGCCGAACTCAC( *Nco*I) |
| | His-rex-Rev | CCCAAGCTTGAAGGCGAGGATCTGGAGCTC (*Hin*dIII) |
| For real-time RT-PCR | | |
| | hrdB-QP-Fw | TACTGCGCAGCCTCAACCAG |
| | hrdB-QP-Rev | GCCGATCTGCTTGAGGTAGTC |
| | hemA-QP-Fw | TCTCTACGTGCACTACGAGG |
| | hemA-QP- Rev | GCGCGTCCTTGATCTGGC |
| | atpI-QP-Fw | CCAAGTGCTTCGCCCTGAC |
| | atpI -QP-Rev | GAGTCCGGCTCCACGTAGAGG |
| | cydA1-QP-Fw | CTCACGCAGGTCTTCCACAC |
| | cydA1-QP-Rev | AGCGAGGTCTTCATCACGGG |
| | nuoA1-QP-Fw | GGAGATGCTGCTCTTCGTGC |
| | nuoA1-QP-Rev | TCAGTCCCATTCCAGACCGC |
| | wblE-QP-Fw | ATGGACTGGCGTCACAACG |
| | wblE-QP-Rev | GGCTTCCTCGATCTGCAGC |
| | aveR-QP-Fw | CAGAAGAACTCACGCTCGTC |
| | aveR-QP-Rev | ACTCTTTCCACAGCCCATTC |
| | aveA1-QP-Fw | CGGACAGGACTACGCACTTC |
| | aveA1-QP-Rev | ACGAGATACGACCGGAGATG |
| | aveD-QP-Fw | GGACTACTACGACCGTTTGACC |
| | aveD-QP-Rev | CTCAGCTTGCCGATGAGGAG |

| Purpose | Primer | Sequence (5'--3')[a] |
|---|---|---|
| | olmRI- QP-Fw | GAGAGAAGGCACACGAGGTC |
| | olmRI- QP-Rev | ATGTCGAGTAAGCGGGAGAG |
| | olmRII- QP-Fw | GGAGGAACTCAGCCTCGACT |
| | olmRII- QP-Rev | ATCTCCCAGCCGATCTTCAC |
| For EMSA and ChIP assay | | |
| | rexp-Fw | GAGTAGTCCAAACAGCCCGG |
| | rexp-Rev | CAGTTGCCACGGTGCTCCT |
| | atpIp-Fw | CGGTGTCATCTCGCTGAGC |
| | atpIp-Rev | GGTGGGCAGAGCACAGTG |
| | cydA1p-Fw | CGTTGTCCAGGTGTTCTACGC |
| | cydA1p-Rev | GGTGGTGATGCCGAACTGC |
| | nuoA1p-Fw | GCTTCATGATCCTTCGCCAG |
| | nuoA1p-Rev | TTCACGCTCCTCGCTCCTC |
| | wblEp-Fw | GAGTCTCTACGTGGCGATCG |
| | wblEp-Rev | TCCATGGCTGCTACCTCTCC |
| | aveRp-Fw | GATGGCCTTCTCCTCCGG |
| | aveRp-Rev | CGTGAGTTCTTCTGGTTTCCG |
| | aveD-A1p-Fw | CCGTCCATCCTCTGCACCTG |
| | aveD-A1p-Rev | GCTGTCGTGGCGATCTACTCC |
| | olmRIp-Fw | CCGCACCCTCCCGACCTG |
| | olmRIp-Rev | GACCTCGCCCAATCAGCACC |
| | olmRIIp-Fw | AATTGCTGGCCGAAGCCC |
| | olmRIIp-Rev | CGAGGACCACACCGCCTT |
| | amfRp-Fw | GGTCAAGGCTCCCTCCTG |
| | amfRp-Rev | CATTGTTGGCGGATGCGAC |
| | amfTp-Fw | GAGCGATTTGAGGGCAAGTT |
| | amfTp-Rev | GTCGGGCGTCTCGTAGAAG |
| | bldMp-Fw | CGGGAAGGCGAGAGAAATCT |
| | bldMp-Rev | CAGACGAGGACGGATGTCA |
| For footprinting assay | | |
| | rexp-FAM-Fw | GAGTAGTCCAAACAGCCCGG |
| | rexp-Rev | CAGTTGCCACGGTGCTCCT |
| | aveRp-FAM-Fw | CCTCTGGACCCTTGCTCG |
| | aveRp-FAM-Rev | CGTGAGTTCTTCTGGTTTCCG |
| | aveD-A1p-FAM-Fw | CCGTCCATCCTCTGCACCTG |
| | aveD-A1p-Rev | GCTGTCGTGGCGATCTACTCC |
| | olmRIp-FAM-Fw | CCGCACCCTCCCGACCTG |
| | olmRIp-Rev | GACCTCGCCCAATCAGCACC |
| | cydA1p-FAM-Fw | CGTTGTCCAGGTGTTCTACGC |
| | cydA1p-Rev | GGTGGTGATGCCGAACTGC |
| | atpIp-FAM-Fw | AGTCCGGGCAGGGTTTCTAC |
| | atpIp-Rev | GGTGGGCAGAGCACAGTG |
| | nuoA1p-FAM-Fw | CTCGGATGCTTCGCGTATGA |

| Purpose | Primer | Sequence (5'--3')[a] |
|---|---|---|
| For 5'RACE assay | nuoA1p-Rev | TTCACGCTCCTCGCTCCTC |
| | oligo (dT)-anchor primer | GACCACGCGTATCGATGTCGACTTTTTTTTTTTTTTTT |
| | sp1-rex | GATCACAACCGGCCAGTCCT |
| | sp2-rex | ACGAGAAGTCCTTGCGCAGC |
| | sp1-aveD | GTGATGCCTCTCAGCTTGCCG |
| | sp2-aveD | TGCGGCCAGTATCCGAGGTG |
| | sp1-aveA1 | GATCATGACGGCGAACAGGGT |
| | sp2-aveA1 | AGTTCCCTTCCCATGCCCGG |
| | sp1-olmRI | TCGTCCGGGAAAGGAGCCC |
| | sp2-olmRI | CAGGATCTCGCTCTTGCCGC |

[a]The restriction enzyme sites are underlined.

## Fig. S1

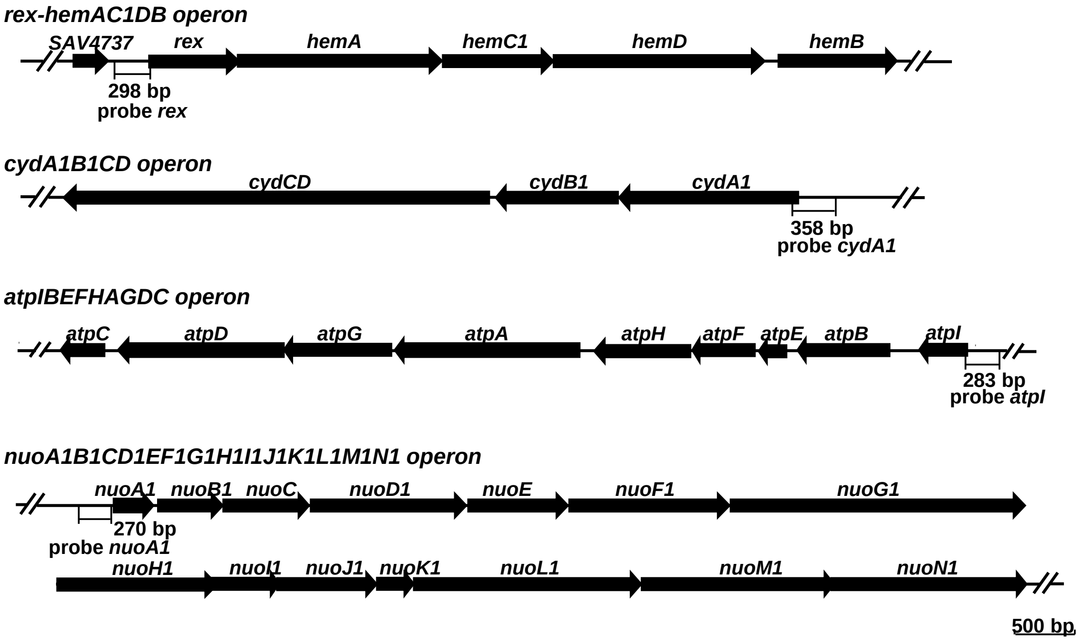


**Fig. S1. Organization of *rex-hemAC1DB*, *cydA1B1CD*, *atpIBEFHAGDC*, and *nuoA1-N1* operons in *S. avermitilis*.** Gene notations are based on the Genome Project of *S. avermitilis* (http://www.ls.kitasato-u.ac.jp/). Lengths and positions of probes used for EMSAs are shown.

## Fig. S2

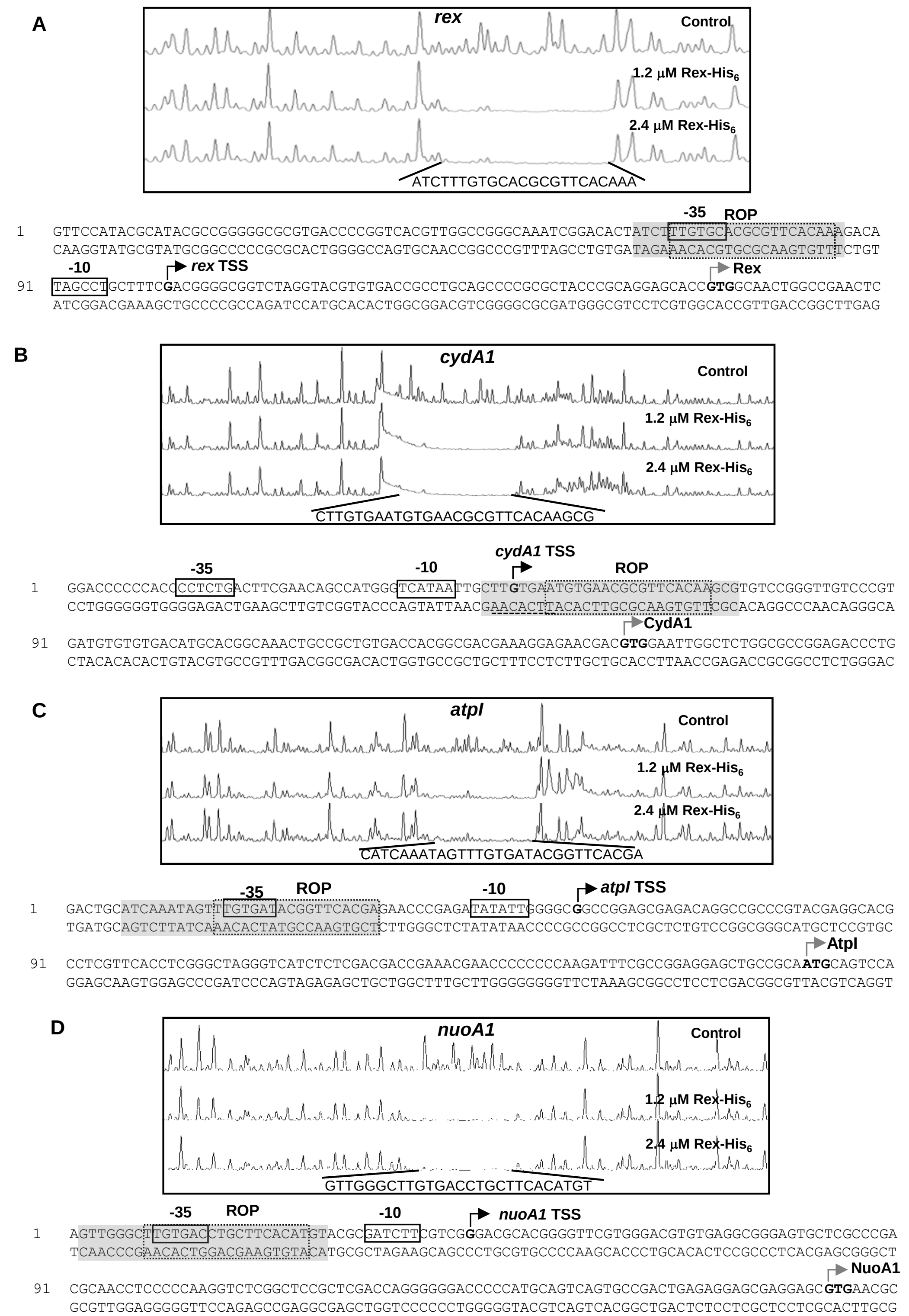


**Fig. S2. Determination of Rex binding sites on promoter regions of *rex* (A), *cydA1* (B), *atpI* (C), and *nuoA1* (D) by DNase I footprinting assay.** Fluorograms correspond to control DNA fragment and to protected reactions (with 1.2 and 2.4 μM Rex-His$_6$). Nucleotide sequences of *rex*, *cydA1*, *atpI*, and *nuoA1* promoter regions are shown below fluorograms. Notations as in Fig. 7.

# Fig. S3

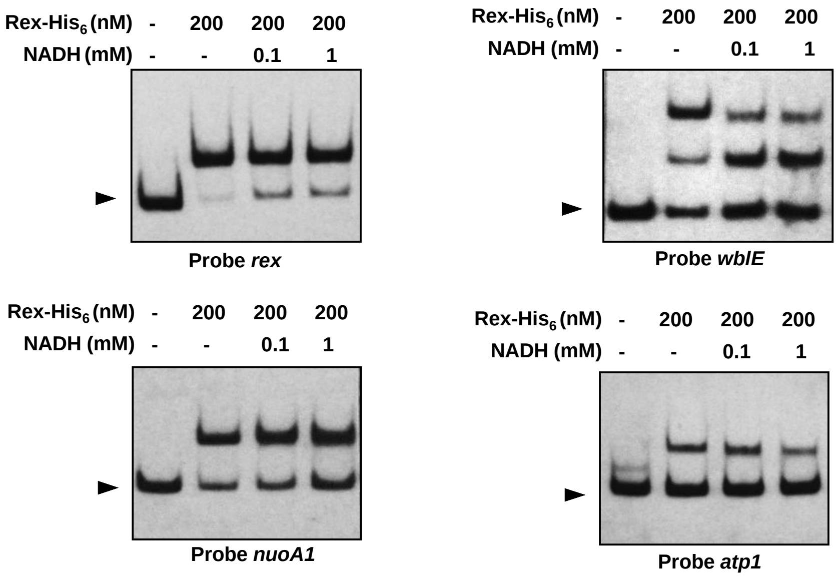


**Fig. S3. DNA-binding activity of Rex is inhibited by NADH.** EMSAs of promoter regions of *rex*, *wblE*, *nuoA1*, and *atpI* using Rex-His$_6$ and NADH. Arrow: free probe.

# Fig. S4

**A**

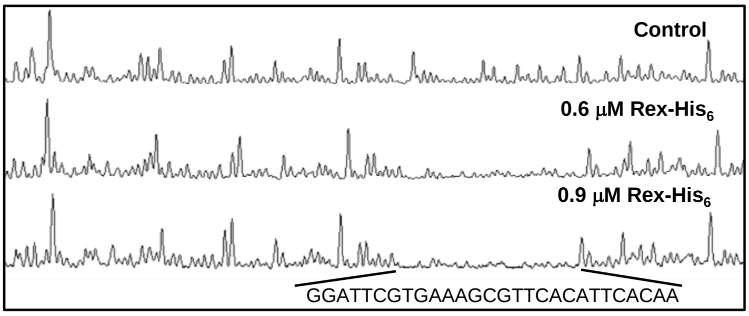


**B**

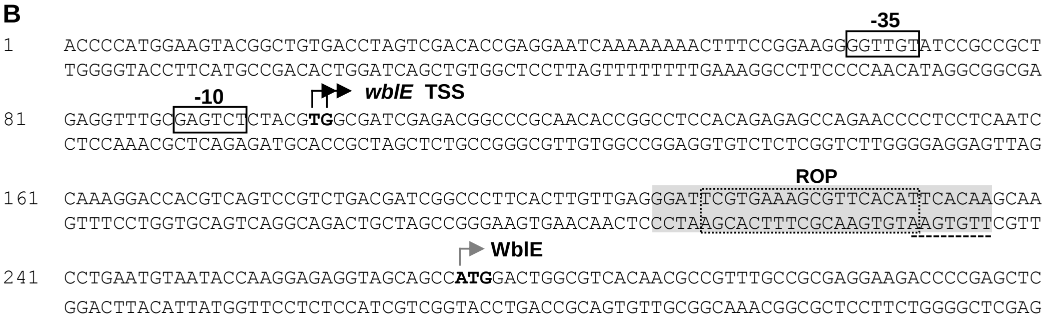


**Fig. S4. Determination of Rex binding site on *wblE* promoter region by DNase I footprinting assay.** (**A**) Fluorograms correspond to control DNA fragment and to protected reactions (with 0.6 and 0.9 μM Rex-His$_6$). (**B**) Nucleotide sequence of *wblE* promoter region. Notations as in Fig. 7.

## Fig. S5

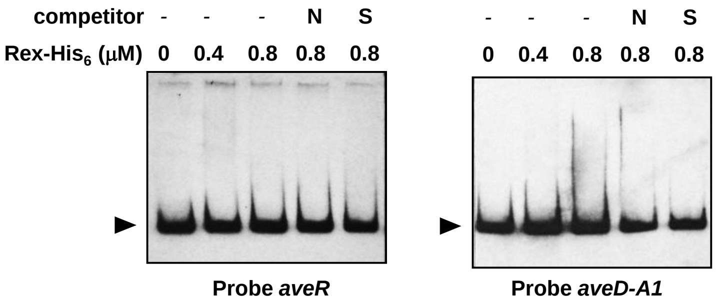


**Fig. S5. Binding of Rex-His$_6$ to *aveR* promoter region and to *aveD-aveA1* intergenic region**. Arrow: free probe. Specificity of band shifts was verified by adding 200-fold excess of unlabeled non-specific competitor DNA (N) and unlabeled specific probe (S).
